# Molecular PET Imaging Versus Conventional Imaging for Detection of Mandibular Metastases in Prostate Cancer: A Paired Retrospective Study

**DOI:** 10.3390/cancers18213436

**Published:** 2026-10-24

**Authors:** Mervat E. Mahmoud, Nur Dizdar, Ahmed E. Ahmed, Eman E. Ahmed, Mindie Mahon, Rimki Haloi, Eugene D. Kwon, Jack Andrews, William Jonathan Fillmore, Ayse T. Kendi

**Affiliations:** 1 Department of Surgery, Division of Oral and Maxillofacial Surgery, Mayo Clinic, Rochester, MN 55905, USA; fillmore.jonathan@mayo.edu; 2 Department of Radiology, Mayo Clinic, Rochester, MN 55905, USA; 3 Department of Surgery, October 6 University Faculty of Medicine, Cairo 12585, Egypt; 4 Department of Urology, Mayo Clinic, Rochester, MN 55905, USA

**Keywords:** neoplasm metastasis, mandible, positron emission tomography, jaw neoplasms, conventional imaging

## Abstract

Purpose: Maxillofacial metastases from prostate cancer account for 9–11% of metastatic jaw lesions. The optimal imaging technique for suspected maxillofacial metastases remains undefined. Conventional imaging often lacks sensitivity for early disease, while PET/CT may be limited due to physiologic tracer uptake in adjacent salivary glands. With increasing use of PET/CT in advanced prostate cancer, its diagnostic performance in the mandibular region requires further evaluation. Methods: We conducted a single-institution paired retrospective study using the prospectively maintained Mayo Clinic Advanced Prostate Cancer Registry. Patients with clinically confirmed mandibular metastases who underwent molecular PET/CT ([^68^Ga]Ga-PSMA-11 or [^11^C]choline) and conventional imaging (CT or MRI) within 1 month were included. All studies were blindly reviewed by a nuclear medicine physician. Detection rates, anatomic distribution, and quantitative imaging parameters were assessed. Paired detection rates were compared using a one-sided exact McNemar test, with a two-sided exact test performed as a sensitivity analysis. Results: Eighteen patients with clinically confirmed mandibular metastases underwent 36 paired imaging evaluations (18 molecular PET/CT and 18 conventional studies). The median age was 70.5 years (IQR, 60–77), and the median Gleason score was 8 (IQR, 7–9). When mandibular metastasis was detected, 56% of our cohort had hormone-sensitive and 44% had castration-resistant disease. Molecular PET/CT detected mandibular involvement in 18/18 patients (100%; 95% CI, 81–100%) versus 13/18 (72.2%; 95% CI, 47–90%) with conventional imaging. All five discordant cases were detected by molecular PET/CT but not conventional imaging, with no discordant cases favoring conventional imaging (one-sided exact McNemar *p* = 0.031; two-sided *p* = 0.063). Mandibular involvement was predominantly unilateral (94.4%), most commonly involving the body (50%), followed by the ramus (27.8%) and condyle (16.6%). The median SUVmax was 6.3 (IQR, 4.4–16.9) for PSMA PET/CT and 4.3 (IQR, 3.8–8.5) for choline PET/CT; the median lesion volume was 5.0 mL (IQR, 1.1–11.3). Conclusions: Molecular PET/CT identified all clinically confirmed mandibular prostate cancer metastases in this paired cohort, including five cases not detected by conventional imaging. Despite physiologic tracer uptake in adjacent salivary structures, mandibular metastatic disease remained detectable on molecular imaging. These findings suggest that molecular PET/CT may provide complementary value when mandibular metastasis is suspected, particularly when conventional imaging is negative or equivocal.

## Simple Summary

Mandibular metastases from prostate cancer are uncommon and can be challenging to detect because symptoms are often nonspecific and imaging interpretation in the maxillofacial region may be difficult. Molecular positron emission tomography/computed tomography (PET/CT), including prostate-specific membrane antigen (PSMA) and choline PET/CT, is increasingly used for evaluation of advanced prostate cancer; however, its performance for detecting mandibular metastases has not been well characterized. In this paired retrospective study, we compared molecular PET/CT with conventional imaging (computed tomography (CT) or magnetic resonance imaging (MRI)) in patients with clinically confirmed mandibular metastases from prostate cancer. Molecular PET/CT detected mandibular metastases in all patients and identified lesions that were not detected by conventional imaging. Despite potential interpretive challenges related to physiologic tracer uptake in adjacent salivary glands, mandibular metastatic disease remained detectable on molecular PET imaging. These findings support the complementary value of molecular PET/CT for detecting mandibular metastases in patients with advanced prostate cancer.

## 1. Introduction

Prostate cancer represents a significant global health burden, with bone being the most common site of distant metastasis at presentation, observed in approximately 82% of patients with de novo metastatic disease [1,2]. Osseous metastases to the axial skeleton are predominant, and involvement of the oral and maxillofacial region is uncommon. Metastatic tumors to the oral cavity account for only 1–3% of all malignant oral lesions, with prostate cancer representing approximately 9–11% of these cases [3,4]. The mandible is more frequently affected than the maxilla or oral soft tissues, with metastases often presenting with nonspecific symptoms including pain, swelling, paresthesia, or numb chin syndrome [5,6,7]. Importantly, oral metastases represent the first indication of metastatic spread or an undiscovered primary malignancy in approximately 23–25% of affected individuals, underscoring the importance of accurate detection and characterization [3,8].

Conventional imaging of suspected osseous metastases has traditionally relied on technetium-99m-methylene diphosphonate (99mTc-MDP) bone scintigraphy in combination with anatomic imaging computed tomography (CT) or magnetic resonance imaging (MRI). However, these modalities demonstrate significant limitations, with pooled sensitivity of bone scan ranging from 79 to 88% and specificity of 75–82% for bone metastasis detection [9,10,11]. The modality’s reliance on osteoblastic activity rather than direct tumor detection results in high rates of equivocal findings (15–25% in large trials) and false-negative examinations, particularly problematic in patients with low prostate-specific antigen (PSA) levels [9,12]. Additionally, plain radiography, while useful for symptomatic regions, cannot detect bone lesions until nearly 50% of mineral content is lost or gained, further limiting its utility in early disease detection [13].

The advent of molecular imaging with prostate-specific membrane antigen (PSMA) and choline positron emission tomography/computed tomography (PET/CT) has revolutionized the diagnostic landscape for prostate cancer staging and restaging. Despite these advances, the interpretation of PSMA and choline PET/CT in the mandibular region presents unique challenges due to intense physiologic tracer uptake in salivary glands and other head and neck structures. PSMA radiotracers demonstrate consistent high uptake in the salivary glands [14,15,16]. Additional pitfalls include PSMA uptake in sympathetic ganglia, benign bone lesions (fractures, osteophytes, and fibrous dysplasia–McCune–albright syndrome), and non-prostatic malignancies, all of which can complicate interpretation in the head and neck region [17,18,19]. The proximity of these sites of physiologic or nonspecific uptake to the mandible may complicate differentiation of metastatic lesions from benign or physiologic findings.

Accurate detection of mandibular metastases has clinical implications beyond disease staging, as these lesions often occur in the setting of widespread metastatic disease and have been associated with a median survival of only 6–8 months following diagnosis [8,20]. Mandibular involvement may also substantially affect quality of life through pain, pathologic fracture, and functional impairment. Current National Comprehensive Cancer Network (NCCN) guidelines incorporate PSMA PET/CT into the evaluation of prostate cancer, including staging of high-risk disease and assessment of biochemical recurrence. Despite the increasing clinical adoption of molecular PET imaging, published experience specifically evaluating its use for mandibular prostate cancer metastases remains limited. This evidence gap is particularly relevant given the potential interpretive challenges created by physiologic tracer uptake in adjacent salivary structures.

Therefore, we evaluated advanced molecular imaging in patients with mandibular metastases from prostate cancer and compared lesion detection with conventional imaging, with particular emphasis on the potential impact of physiologic salivary gland uptake on lesion identification.

## 2. Methods

### 2.1. Study Design and Patient Population

We conducted a single-institution paired retrospective study from 2008 to 2024 using a maintained advanced prostate cancer registry. Patients with advanced prostate cancer and clinically confirmed mandibular metastases who underwent a paired radiographic evaluation with PSMA PET/CT or choline PET/CT and conventional imaging (CT or MRI) within 1 month of each other were included. Among the molecular imaging studies, 9 patients underwent 68Ga-PSMA-11 PET/CT and 9 underwent 11C-choline PET/CT. Among conventional imaging studies, 13 patients underwent CT and 5 underwent MRI.

Mandibular metastases were confirmed using a predefined composite clinical–radiographic reference standard incorporating radiographic findings on multiple imaging modalities, PSA levels, presence of metastatic disease at other sites, radiographic progression on serial imaging, longitudinal clinical follow-up, and/or response to systemic therapy. Histopathologic confirmation was available in 3 of 18 patients (17%); however, all cases were adjudicated using the same composite reference framework. Histopathology confirmation was obtained according to clinical judgment. The study protocol was approved by the Institutional Review Board, and all patient data were de-identified in accordance with institutional and regulatory requirements before analysis.

### 2.2. Imaging Acquisition

Conventional imaging included CT and/or MRI of the head and neck, while molecular imaging included [^68^Ga]Ga-PSMA-11 PET/CT or [^11^C]choline PET/CT scans. For PSMA PET/CT, imaging was performed on digital PET/CT scanners (Discovery; GE Healthcare (Chicago, IL, USA); Biograph Vision 600 and Biograph Vision Quadra Edge; Siemens Healthineers). All scanners underwent routine physicist-led calibration, harmonization, and quality assurance procedures to ensure internal consistency of standardized uptake value (SUV) measurements. Patients received 270 ± 70 MBq intravenously, followed by an uptake period of approximately 60 min. A low-dose non-contrast CT scan from the vertex or orbits to the upper thighs was acquired for attenuation correction and anatomic co-registration.

For ^11^C-choline PET/CT, studies were performed according to institutional clinical protocols using PET/CT systems (Discovery LS, RX, 690, or 710; GE Healthcare). Patients received 370–555 MBq of ^11^C-choline intravenously, and imaging began approximately 5 min after injection. PET images were acquired from the orbits to the mid-thigh and reconstructed using a three-dimensional ordered-subsets expectation maximization algorithm (28 subsets; two iterations). Low-dose CT images were obtained for attenuation correction and anatomic localization. All imaging studies were independently and blindly reviewed by a nuclear medicine physician. Readers were blinded to clinical information except for prostate cancer diagnosis and were blinded to the results of other imaging modalities. Because all images were reviewed by a single reader, inter-reader agreement was not assessed. For each modality, presence or absence of mandibular metastases was recorded per patient. Anatomic location, lesion characteristics, and laterality of involvement were documented. Multifocal disease was defined as involvement of more than one mandibular anatomical site in the same patient and was analyzed as a separate category. Quantitative PET/CT analysis included SUVmax and metabolic lesion volume. Lesions were manually segmented by the nuclear medicine physician using MIM software (7.3.5 version, MIM Software Inc., Cleveland, OH, USA), and quantitative measurements were recorded for analysis. For conventional imaging, lesion size and morphologic features were measured.

### 2.3. Statistical Analysis

Descriptive statistics were performed using JMP version 18 to summarize patient demographics, clinical characteristics, and lesion distribution. Continuous variables, including age and SUVmax values, were reported as median and interquartile range (IQR), while categorical variables were expressed as counts and percentages. The detection rates of mandibular metastases by PSMA PET/CT, choline PET/CT, CT, and MRI were compared. Differences in quantitative PET/CT parameters between PSMA and choline tracers were assessed. A *p*-value < 0.05 was considered statistically significant.

Because all included patients had clinically confirmed mandibular metastases, the analysis compared the relative detection performance of the imaging modalities rather than overall diagnostic accuracy. Accordingly, specificity, false-positive rates, and predictive values could not be assessed. Because of the rarity of mandibular metastatic disease, PSMA and choline PET/CT examinations were grouped as molecular PET imaging for the primary analysis. The study was not powered for tracer-specific comparisons. Detection rates of advanced PET/CT and conventional imaging were compared using an exact one-tailed McNemar test for paired data. A one-tailed test was prespecified because extensive evidence indicates that PSMA- and choline-based PET/CT are more sensitive than conventional imaging for detecting bone metastases, making superior performance of conventional imaging biologically unlikely. A two-sided exact McNemar test was additionally performed as a sensitivity analysis.

## 3. Results

### 3.1. Patient Characteristics

The median age at diagnosis was 70.5 years (interquartile range [IQR], 60–77), and the median [IQR] primary Gleason score was 8 (7–9). Nearly half of the patients presented with metastatic disease at initial diagnosis and received upfront chemohormonal therapy, whereas the remaining patients developed metastatic progression following initial local treatment. At the time of detection of mandibular metastases, 56% of patients had hormone-sensitive disease, while 44% had castration-resistant prostate cancer [see Table 1].

### 3.2. Imaging Analysis and Detection Rates

Eighteen patients with clinically confirmed mandibular metastases were included. Each patient underwent paired molecular PET imaging and conventional imaging, yielding 36 imaging studies for analysis, including 18 PET/CT scans (PSMA PET/CT ([^68^Ga]Ga-PSMA-11) or choline PET/CT ([^11^C]choline)) and 18 conventional imaging studies (CT or MRI). Advanced PET/CT imaging detected mandibular metastases in 100% (95% CI, 81–100%) of cases, compared with 73% (95% CI, 47–90%) by conventional imaging (one-tailed McNemar exact test, *p* = 0.031). Among five discordant cases, all were detected by PET/CT but missed by conventional imaging; no cases were detected by conventional imaging alone [see Table 2]. Among the conventional imaging modalities (CT or MRI) failed to identify mandibular metastatic involvement in 27% of cases that were subsequently detected on advanced PET/CT imaging. In cases where both modalities identified the lesion, advanced PET/CT imaging provided additional information regarding metabolic activity and tracer avidity that was not available on conventional imaging.

### 3.3. Anatomic Distribution of Mandibular Metastases

**Figure 1:**
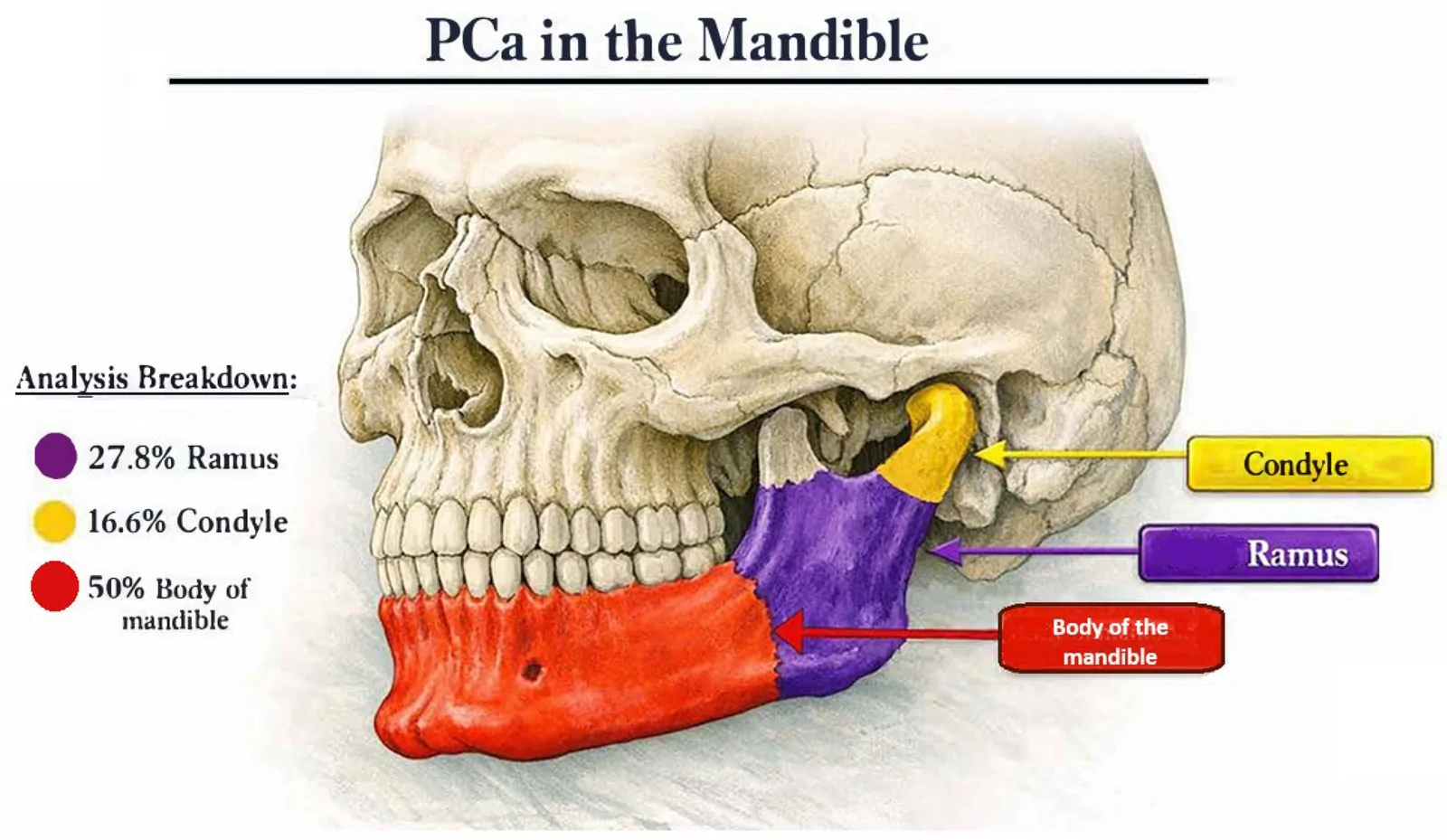
Distribution of metastatic mandibular lesion. Distribution of mandibular metastases from prostate cancer. The mandibular body was the most frequently involved site (50%), followed by the ramus (27.8%), condyle (16.6%) and multifocal (5.6%).

The majority of lesions were unilateral (94.4%), with only 5.6% demonstrating bilateral involvement. The mandibular body was the most commonly involved anatomic site, accounting for 50% of all mandibular metastases. The ramus and the condyle were involved in 27.8% and 16.6% of cases, respectively, while multifocal involvement was present in 5.6% of cases. No lesions were identified in the maxilla or other facial bones in this cohort [see Table 3; Figure 1].

### 3.4. Quantitative PET/CT Imaging Parameters

Quantitative analysis of tracer uptake revealed differences between PSMA PET/CT and choline PET/CT imaging. The median (IQR) SUVmax of mandibular lesions on PSMA PET/CT was 6.3 (4.4–16.9), demonstrating higher tracer avidity compared to choline PET/CT, which showed a median (IQR) SUVmax of 4.3 (3.8–8.5). The median (IQR) lesion volume was 6.8 mL (1.2–19.4) of PSMA PET/CT and the median (IQR) lesion volume was 1.8 mL (0.9–8.8) of choline PET/CT [see Table 4].

## 4. Discussion

In this paired retrospective study, molecular PET/CT demonstrated a higher detection rate for clinically confirmed mandibular prostate cancer metastases than conventional imaging. Molecular PET/CT identified mandibular involvement in all 18 patients (100%), compared with 13 of 18 patients (72.2%) using conventional CT or MRI. Importantly, all five discordant cases were detected by molecular PET/CT but not by conventional imaging, with no discordant cases favoring conventional imaging. These findings are particularly relevant given the rarity of mandibular prostate cancer metastases and the potential interpretive challenges posed by physiologic tracer uptake in adjacent salivary and other head and neck structures.

Metastatic tumors involving the oral and maxillofacial region are rare, accounting for approximately 1% of all malignant oral neoplasms [21]. Among metastatic lesions involving the jaws, prostate carcinoma accounts for approximately 9–11% of cases, making it one of the more frequent primary malignancies metastasizing to the jaw in male patients [3,4]. Within the maxillofacial skeleton, the mandible is the most commonly affected site, followed by the maxilla, with a particular predilection for the posterior mandibular region [8]. The predominance of mandibular body and ramus involvement observed in the present cohort is consistent with previously described patterns of metastatic spread to the jaw. Several anatomical factors have been proposed to explain this distribution, including the relatively greater amount of hematopoietically active marrow and vascular supply within the posterior mandible. Recognition of these characteristic patterns may be clinically relevant, as mandibular metastatic disease often presents with nonspecific symptoms that can mimic benign odontogenic or temporomandibular disorders, potentially delaying diagnosis and appropriate oncologic evaluation [22,23]. Awareness of these patterns may therefore facilitate consideration of metastatic disease in patients with advanced prostate cancer presenting with new mandibular symptoms. Despite these recognized patterns, evidence comparing molecular PET/CT with conventional imaging specifically for mandibular prostate cancer metastases remains limited and has largely consisted of case reports and small case series. Our paired cohort therefore provides a more structured assessment of the relative detection of mandibular metastatic involvement using molecular and conventional imaging.

These results are consistent with the broader evidence supporting improved detection of prostate cancer metastases using molecular imaging. A meta-analysis comparing 68Ga-PSMA-11 PET/CT with 99mTc-MDP bone scintigraphy reported pooled sensitivity and specificity of 98% and 97% for PSMA PET/CT versus 83% and 68% for bone scintigraphy [24]. Similarly, the proPSMA trial demonstrated that PSMA PET/CT offers 27% greater accuracy than conventional imaging for detecting metastatic disease in high-risk patients [25]. Additional systematic reviews have confirmed that PSMA PET/CT significantly outperforms CT, MRI, and bone scintigraphy for detecting bone metastases, with accuracy values of 0.95 versus 0.85, 0.81 and 0.80 [26,27]. PSMA PET/CT may also identify osseous metastatic disease in patients with negative bone scintigraphy, whereas bone scintigraphy appears to provide limited additional diagnostic value following negative PSMA PET/CT findings [24,28]. Choline PET/CT has similarly demonstrated high diagnostic performance for detecting osseous metastases, with reported pooled sensitivity of 89–91% and specificity of 98–99% [12,29]. Although these studies establish the broader diagnostic advantages of molecular imaging for osseous metastatic disease, the present study extends these observations to the mandible, an anatomically distinct and potentially challenging region.

The mandibular region presents unique challenges for molecular PET interpretation because of physiologic tracer uptake in adjacent head and neck structures, particularly the salivary glands. The parotid and submandibular glands demonstrate consistently high tracer uptake, with reported mean SUVmax values of 12.3 and 11.7, respectively [14]. Furthermore, intense radioligand accumulation in salivary tissue does not appear to correlate with high PSMA expression on immunohistochemistry, suggesting that salivary uptake may occur through mechanisms that are not entirely PSMA-mediated [30]. Other potential interpretive pitfalls include tracer accumulation in sympathetic ganglia, inflammatory processes, and benign osseous abnormalities. Despite these potential sources of interference, molecular PET/CT identified all clinically confirmed mandibular metastases in our cohort. The unidirectional discordance observed in the five patients with negative conventional imaging is consistent with the ability of molecular imaging to identify tracer-avid metastatic deposits that may lack conspicuous structural abnormalities on CT or MRI. Importantly, however, our findings should not be interpreted as demonstrating earlier detection, as the paired imaging examinations were obtained within the same clinical interval.

From a clinical perspective, accurate recognition of mandibular metastatic disease may influence disease staging, symptom attribution, and treatment planning. In patients presenting with mandibular pain, swelling, or numb chin syndrome, advanced molecular imaging may facilitate identification of metastatic disease when conventional imaging findings are inconclusive. Given that all discordant cases in the present cohort favored molecular imaging, PET/CT may be a useful adjunct when mandibular metastatic disease is suspected, and conventional imaging findings are negative or equivocal.

To our knowledge, this represents one of the first studies comparing PSMA or choline PET/CT with conventional imaging for the detection of mandibular prostate cancer metastases. The paired design, in which each patient underwent both molecular and conventional imaging within 1 month, allowed direct within-patient comparison and represents an important strength given the rarity of this metastatic presentation. In addition, multidisciplinary evaluation incorporating expertise from nuclear medicine, radiology, oral and maxillofacial surgery, and oncology enabled characterization of anatomic distribution, lesion features, and quantitative PET/CT parameters. Together, these findings provide preliminary evidence supporting the complementary clinical utility of molecular PET/CT in the evaluation of suspected mandibular metastatic involvement.

Our study has several limitations. First, its retrospective design and single-institution setting may limit the generalizability of our findings, although the small cohort size reflects the rarity of mandibular metastases from prostate cancer. Information regarding imaging-related changes in staging, treatment planning, or clinical management was not systematically collected and therefore could not be evaluated in the current study. Second, molecular imaging included both PSMA and choline PET/CT, while conventional imaging included CT and MRI, introducing heterogeneity within the imaging groups. The limited sample size precluded formal tracer-specific comparisons, and PSMA- and choline-specific quantitative findings should therefore be considered descriptive. Third, because all included patients had clinically confirmed mandibular metastatic disease, the study evaluated relative detection rates rather than overall diagnostic accuracy; specificity, false-positive rates and predictive values could not be assessed due to the small sample size, which reflects the rarity of the condition. Histopathologic confirmation was not available for all lesions, and the detection of mandibular metastasis was instead established through integrated clinical, laboratory, longitudinal, and imaging assessment. This composite reference standard may introduce potential incorporation bias because imaging findings contributed to the clinical determination of metastatic involvement. In addition, a subgroup analysis restricted to histopathologically confirmed cases was not performed because of the limited number of biopsy-confirmed cases, which may affect the stability of statistical analysis. Finally, the relatively small number of discordant pairs limits statistical precision despite the consistent direction of discordance and restricts the ability to perform subgroup analyses. Larger prospective and multicenter studies incorporating standardized imaging protocols and, when feasible, histopathologic confirmation are warranted to validate these findings and better define the diagnostic performance of molecular PET/CT for mandibular prostate cancer metastases, in addition to evaluating its impact on staging, treatment planning, and clinical management. Future studies with larger cohorts may permit more robust comparisons between individual PET tracers and conventional imaging modalities.

## 5. Conclusions

Advanced molecular imaging identified mandibular metastatic lesions not visualized by conventional imaging and remained effective despite the potential interpretive challenge of physiologic salivary gland uptake. All discordant findings favored molecular imaging, with no lesion detected exclusively by conventional imaging. These findings support incorporation of molecular PET imaging into the evaluation of suspected mandibular metastatic lesions in patients with advanced prostate cancer. Improved detection of mandibular metastases may facilitate diagnosis and subsequent clinical decision-making. Future studies are warranted to further evaluate the impact of molecular PET imaging on staging, treatment planning, and clinical management.

## Figures and Tables

**Table 1:** Patient demographics and characteristics.

Patient Variables	Value
Median (IQR) age at diagnosis	70.5 years (60–77)
Median (IQR) primary Gleason score	8 (7–9)
Metastatic disease at initial diagnosis	9 (50%)
Upfront treatment in metastatic cases	Chemohormonal therapy
Hormone-sensitive disease at mandibular metastasis detection	10 (56%)

**Table 2:** Imaging studies and detection rates.

Imaging Modality	Number of Studies	Detection of Mandibular Metastases	*p*-Value
PSMA PET/CT or choline PET/CT	18	18 (100%)	0.031
Conventional CT or MRI	18	13 (73%)	0.031
Total imaging studies reviewed	36	—	—

**Table 3:** Distribution of metastatic mandibular lesions.

Location	N (%)
Unilateral lesions	17 (94.4%)
Bilateral lesions	1 (5.6%)
Body of mandible	9 (50%)
Ramus	5 (27.8%)
Condyle	3 (16.6%)
Multifocal	1 (5.6%)

**Table 4:** PET/CT imaging characteristics.

Parameter	PSMA PET/CT	Choline PET/CT
Median (IQR) SUVmax	6.3 (4.4–16.9)	4.3 (3.8–8.5)
Median (IQR) lesion size (all lesions)	6.8 mL (1.2–19.4)	1.8 mL (0.9–8.8)

## Data Availability

The original contributions presented in this study are included in the article. Further inquiries can be directed to the corresponding authors.
